# Synthesis and Properties of Cationic Core-Shell Fluorinated Polyurethane Acrylate

**DOI:** 10.3390/polym16010086

**Published:** 2023-12-27

**Authors:** Junhua Chen, Xiaoting Lu, Jinlian Chen, Shiting Li, He Zhang, Yinping Wu, Dongyu Zhu, Xiangying Hao

**Affiliations:** 1 School of Environmental and Chemical Engineering, Zhaoqing University, Zhaoqing 526061, China; cehjchen@yeah.net (J.C.); luxiaoting23@mails.ucas.ac.cn (X.L.);; 2 Guangdong Provincial Key Laboratory of Environmental Health and Land Resource, College of Environmental and Chemical Engineering, Zhaoqing University, Zhaoqing 526061, China; 3 School of Chemical Engineering and Light Industry, Guangdong University of Technology, Guangzhou 510006, China; zdy16@gdut.edu.cn

**Keywords:** cationic waterborne polyurethane, core-shell structure, isocyanate reactivity, tensile strength, acrylate-based composites

## Abstract

Vinyl-capped cationic waterborne polyurethane (CWPU) was prepared using isophorone diisocyanate (IPDI), polycarbonate diol (PCDL), *N*-methyldiethanolamine (MDEA), and trimethylolpropane (TMP) as raw materials and hydroxyethyl methacrylate (HEMA) as a capping agent. Then, a crosslinked FPUA composite emulsion with polyurethane (PU) as the shell and fluorinated acrylate (PA) as the core was prepared by core-shell emulsion polymerization with CWPU as the seed emulsion, together with dodecafluoroheptyl methacrylate (DFMA), diacetone acrylamide (DAAM), and methyl methacrylate (MMA). The effects of the core-shell ratio of PA/PU on the surface properties, mechanical properties, and heat resistance of FPUA emulsions and films were investigated. The results showed that when w(PA) = 30~50%, the stability of FPUA emulsion was the highest, and the particles showed a core-shell structure with bright and dark intersections under TEM. When w(PA) = 30%, the tensile strength reached 23.35 ± 0.08 MPa. When w(PA) = 50%, the fluorine content on the surface of the coating film was 14.75% and the contact angle was as high as 98.5°, which showed good hydrophobicity; the surface flatness of the film was observed under AFM. It is found that the tensile strength of the film increases and then decreases with the increase in the core-shell ratio and the heat resistance of the FPUA film is gradually increased. The FPUA film has excellent properties such as good impact resistance, high flexibility, high adhesion, and corrosion resistance.

## 1. Introduction

The substantial environmental degradation that has been occurring in recent years has focused a lot of emphasis on the study and development of environmentally friendly materials [1,2,3]. Waterborne polymers have been employed extensively in recent decades due to their low toxicity and environmental friendliness [4,5,6]. Waterborne resins have hydrophilic groups with high surface energy but low water and solvent resistance; waterborne polyurethanes are an important family of ecologically benign waterborne resins with good mechanical and physical properties [7,8]. However, cationic aqueous polyurethanes with sulfur or ammonium ions in the side chain or main chain have a strong hygroscopicity and bactericidal activity and they are frequently used to treat surfaces made of paper, leather, cloth, and glass [9]. Acrylates have good water resistance and double bonding aids in the formation of a crosslinked structure; therefore, combining them with aqueous polyurethanes can result in composite emulsions with outstanding overall performance [10]. On the other hand, the mechanical properties of single polyurethane aqueous dispersions are poor, with poor self-thickening and low solid content. Some of the promising applications of aqueous polyurethanes formed using isocyanate chemistry are electrode functionalization for electrochemical measurements [11], plant-based cellulose polymers for single-molecule studies [12], or metal oxide surface conjugation as a suitable chemical platform [13].

As a result of their strong electronegativity and low polarization, fluorine atoms can effectively lower the surface tension of polymers, allowing them to display a variety of benefits like stability, good weathering, and resistance to heat [14]. Perfluoroalkyl acrylic acid copolymers, for example, have an extremely low critical surface tension in the range of 10–11 mN/m [15]. This is because fluorocarbon side-chain-containing polymers have a high abundance of CF_3_ groups on the surface. When fluorine atoms are added to polyurethane acrylate systems, they can effectively reduce their surface energy and improve their thermal stability and excellent water resistance [16,17,18,19,20]. Fluorine atoms have unique properties, which makes fluorinated polymers outstanding and unique [21]. Nevertheless, fluorinated monomers are not very compatible and come at a hefty price [22]. Copolymer compatibility and emulsion stability are known to diminish with higher incorporation of fluorinated monomers in copolymers [23]. Consequently, it is important to optimize the polymerization process to minimize the number of fluorinated monomers while preserving a suitable surface tension (oil/water repellency).

Due to their general benefits in film production, core-shell fluorinated copolymer emulsions have drawn a lot of interest when compared to regular emulsions without unique features [24,25,26,27]. The construction of the core-shell structure allows the acquisition of final polymer properties that are difficult to obtain by blending or random copolymerization of the two polymers [28,29,30]. It also improves the copolymer properties such as impact resistance, abrasion resistance, and water resistance because of the ionic bonding, grafting, or interpenetrating networks between the core-shell layers [31,32]. Typically, semi-continuous or seeded emulsion polymerization is used to create core-shell fluorinated copolymers [33]. This process involves polymerizing a mixture of monomers to form a nucleus and then post-polymerizing a different mixture of monomers on the nucleus seed to generate a shell [34]. The core-shell technique can be categorized as either copolymerized or non-copolymerized depending on how the PU and FPA chain breaks are connected.

By employing a monomer pre-emulsification synthesis technique, Zhong added hexafluorobutyl methacrylate (HFMA) to waterborne polyurethane dispersion and created crosslinked fluorinated polyurethane resin (FWPU) with a uniform particle size by using dihydrazide adipate as a chain extender [35]. By esterifying perfluorooctane chloride (PFOC) and hydroxypropyl methacrylate (HPMA), Bai created the fluorine-containing acrylate monomer PFMA [36]. Potassium persulfate (KPS) and sodium bicarbonate were then utilized as an initiator/buffer system, while sodium dodecyl sulfate (SDS)/Tween 80 was used as a mixed emulsifier. Semi-continuous core-shell polymerization was used to create the fluorinated acrylate emulsions MMA/BA/St and PFMA/MMA/BA. Ting used methyl methacrylate (MMA), butyl acrylate (BA), dodecafluoroheptyl methacrylate (DFMA), and 3-(Trimethoxysilyl)propyl methacrylate (MPS) as raw materials to create a series of self-crosslinked fluorinated polyacrylate emulsion particles with core-shell structure [37]. Investigations were performed into how the MPS dose affected the characteristics of latex film. By using the core-shell polymerization process, Hirose created a polyurethane/polyacrylate composite emulsion with an interpenetrating polymer network (IPN) [38]. By combining vinyl end-sealed polyurethane prepolymer and a combination of acrylate and perfluoroalkyl acrylate as raw ingredients, Park created a water-based fluorinated polyurethane-acrylate emulsion [14]. It was investigated as to how much PA/FPA affected the surface, thermal, and mechanical characteristics of polymer antifouling coatings. In their study, Chakrabarty prepared polystyrene nuclear emulsion particles with perfluoroalkyl acrylate as the shell layer [39]. They demonstrated that, when compared to random copolymers or emulsion blends of styrene and perfluoroalkyl acrylate, coil-shell particles are the most effective particles for lowering the surface energy of emulsion film.

Certain desired features of polyacrylate coatings can be introduced by adding fluorine-containing groups to copolymers in polyurethane acrylics [40]. Consequently, high-end self-cleaning coatings and water-/oil-repellent surface coatings for textiles, papers, and leathers have been using more and more fluorinated polyurethane-acrylate (FPUA) [41]. It has also progressively developed into a focal point for domestic and international research on fluorine-modified waterborne polyurethanes [42]. On the other hand, cationic fluorinated waterborne polyurethanes have not been the subject of as many national and international investigations. In addition, there are many accessible fluorinated monomers but their utilization rate is low, the polymerization process is hard to manage, and the reaction process is complicated.

In this study, cationic FPUA that self-emulsifies was created by adding unsaturated monomer to partially seal the PU chain break that contains an NCO end group. By combining this process with monomer pre-emulsification, hydrophobic PA/FPA monomer was expanded from the outside to the inside of PU micellar for polymerization. The polymerization process was straightforward and simple to manage. Transmission electron microscopy (TEM), a contact angle measurement device, infrared spectroscopy, and XPS were used to describe and compare the structure and surface hydrophobic characteristics of PU and modified FPUA.

## 2. Materials and Methods

### 2.1. Materials

Polycarbonate diol (PCDL, purity > 95%, M_n_ ~ 1000) was procured from Shanghai Huihua Industrial Co., Ltd., Shanghai, China. Isophorone diisocyanate (IPDI, purity > 99.5%, NCO% ≥ 37.5%) was supplied by Bayer Chemicals (Leverkusen, Germany). Dodecafluoroheptyl methacrylate was purchased from Shangfu Technology Co., Ltd., Shanghai, China. *N*-methyldiethanolamine (MDEA, ≥99.5%), 1,4-butanediol (BDO, ≥99.5%), methyl methacrylate (MMA, ≥99.5%), hydroxyethyl methacrylate (HEMA, ≥96%), trimethylolpropane (TMP, ≥99.5%), and dibutyltin dilaurate (DBTDL, ≥99.5%) were purchased from Macklin Inc., Shanghai, China. Dihydrazide adipate (ADH, ≥98%) and 2,6-tert-butyl-*p*-cresol (BHT, ≥99.5%) was supplied by Aladdin Reagent Co., Ltd., Shanghai, China. Glacial acetic acid (HAc, ≥90%) and ammonium persulfate (APS, ≥99%) were purchased from Shanghai Lingfeng Chemical Reagent Co., Ltd., Shanghai, China. Deionization is homemade in the laboratory. All the other reagents underwent further purification.

### 2.2. Preparation of Cationic Polyurethane Aqueous Dispersion (CWPU)

**Scheme 1:**
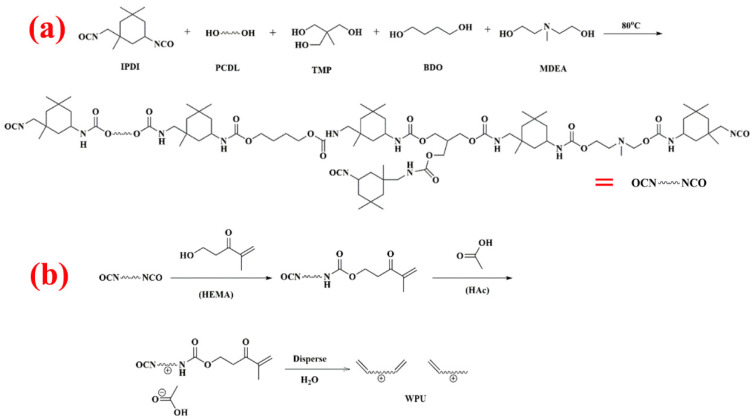
Synthesis of the CWPU. (**a**) Isocyanate-terminated prepolymers; (**b**) Cationic polyurethane aqueous dispersion.

Pre-add PCDL and IPDI in a three-necked flask with a stirrer, raise the temperature to 80~85 °C, drop in catalyst DBTDL for 1~2 h, and then add TMP and BDO to keep the reaction for 2 h. When the viscosity of the system rises, inject an appropriate amount of acetone to adjust the viscosity of the system. After the system is stabilized, the temperature of the reaction system is lowered to 60 °C, and then MDEA is added. In order to reduce the viscosity of the system and to reduce the foam in the synthesis, the system is diluted by adding an appropriate amount of acetone and the acceleration of the drop is adjusted so that it is all added within one hour. The heating is then continued for two to three hours. HEMA is added to the system, the ends are blocked with double bonds, held for two hours, and then completely neutralized with glacial acetic acid. Finally, an amount of deionized water is gradually added to the prepolymer and emulsified with rapid shear. This resulted in a cationic partially double-bonded aqueous polyurethane dispersion. The crude reaction mixture was at a 75% yield. Scheme 1 is shown in the figure below, with the detailed dosages shown in Table S1.

### 2.3. Preparation of Fluorinated Waterborne Polyurethane-Acrylate Core-Shell Emulsion (FPUA)

**Scheme 2:**
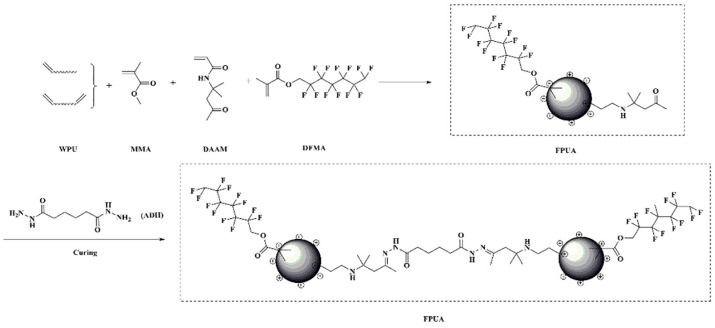
Preparation of FPUA.

As one of the reaction monomers, the above-obtained polyurethane dispersion was added together with MMA, DAAM, and DFMA, each of which accounted for 30% and 50% of the total weight. DFMA made up 10% of the total weight. To create FPUA, cored shell emulsions with cored shells of 30/70 and 50/50 were used as well as 0.2 g APS initiator, which was introduced for the emulsion polymerization reaction after an even mixing step at 80 °C. The temperature was then maintained for 4 h. The crude reaction mixture was at 86% yield. In Table S1, the typical formulations are displayed. The synthetic circuit is shown in Scheme 2.

### 2.4. Preparation of FPUA Film

Place the emulsion in a Teflon mold and allow it to cure for a week at room temperature before putting it in a vacuum drying oven at 60 °C for 72 h to produce a light-yellow film with a thickness of around 1 mm.

### 2.5. Characterization

A Fourier transform infrared spectrometer (PERKIN ELMER 1730, Waltham, MA, USA) was used to conduct FTIR tests using the potassium bromide tablet method. The range of wave numbers is 400–4000 cm^−1^. The membranes were prepared using a Fourier Transform Attenuated Total Reflectance Infrared Spectrometer (ATR-FTIR) manufactured by Nicolet Company, Madison, WI, USA, with a wave number range of 4000–525 cm^−1^ and 32 scans with a resolution of 4 cm^−1^. At room temperature, the emulsion was diluted 1000 times and then the particle size and distribution of the emulsion were measured by Zetasizer Nano-ZS (Malvern, UK). The UJC-2000C1 static contact angle measuring instrument (Shanghai Zhongchen Digital Equipment Co., Ltd., Shanghai, China) measured the contact angles of the surface layer of the coating film and the air medium at room temperature with distilled water, dichloroethane, and hexadecane. The emulsion was diluted 200 times with deionized water and then thoroughly mixed using a sonicator. It was then dripped onto a conductive copper mesh and stained with 2% phosphotungstic acid and, after drying, the morphology of the stained emulsion particles was examined using a transmission electron microscope (TEM, Hitachi, Japan, model H-7650) with an operating voltage of 60 kV. The samples to be tested were uniformly coated on the surface of monocrystalline silicon, their surfaces were sprayed with gold after drying, and they were then characterized in a scanning electron microscope (SEM) model SU-8220 of Hitachi, Japan. The samples were uniformly coated on the monocrystalline silicon surface before testing and the samples were tested with a SPA-400 knockdown atomic force microscope (AFM) with a scanning range of 5 × 5 μm and a viewing scale of 10 nm. Each sample was finally presented in the form of a two-dimensional planar image as well as a three-dimensional image. A Hitachi GENESYS 180 UV–visible spectrometer was used to analyze the transmittance of the coatings. A blank slide served as the test backdrop, while a slide served as the substrate. After testing each sample group three times in parallel at three separate test sites, the average transmittance at 500 nm for the three groups was determined.

The film was heated at 80 °C for 2 h and then the surface elements of the film were analyzed qualitatively or quantitatively by using an X-ray photoelectron spectrometer (ESCALAB 250XI, Thermo Scientific, Waltham, MA, USA). The heat loss of the samples was measured using a thermogravimetric differential thermal integrated analyzer (Labsys Evo) from Setaram, Caluire-et-Cuire, France, with temperatures ranging from ambient temperature to 600 °C with an increasing rate of 10 °C/min. A dynamic thermo-mechanical analyzer model DMA 242C3 from NETZSCH, Selb, Germany, was used to test the thermo-mechanical properties of the coatings. The following parameters were used: maximum amplitude of 10 μm, maximum dynamic force of 2 N, static force of 0.5 N, temperature scanning range of −50~100 °C, temperature rise rate of 5.0 K/min, test frequencies of 1.0 Hz, 3.333 Hz, and 5.0 Hz. A multifunctional electronic tensile testing machine EKT-TS2000 (Ektron Tek Co., Ltd., Taiwan, China) was used to measure the mechanical behavior of the sample. The tensile test speed was 10 mm/min. Specimens with specifications of 80 mm × 10 mm (length × width) were used for the evaluation. The samples were cut into tensile test strips of 0.5–1.0 mm thickness. The test was repeated three times to ensure the accuracy of the measurement results. To test the corrosion resistance of the coated film, four different solutions were used: 5.0 wt% NaCl solution, 0.5 mol/L CuSO_4_ solution, H_2_SO_4_ solution (pH = 0, using methyl orange staining), and NaOH solution (pH = 14, using rhodamine staining). Then, 0.5 mL droplets were taken and added to the coated and uncoated areas, respectively, and the corrosion of the respective surfaces was observed after 24 h. The corrosion of the coated and uncoated surfaces was observed after 24 h.

## 3. Results and Discussion

**Figure 1:**
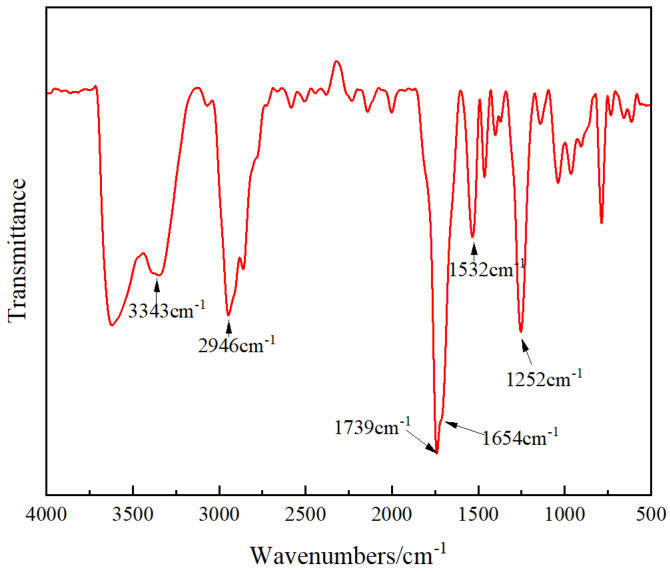
FT-IR spectrum of CWPU.

The stretching vibration absorption peak of –NH– and –OH is at 3343 cm^−1^ in Figure 1. The characteristic absorption peak of –CH_3_ and –CH_2_– is at 2946 cm^−1^, the stretching vibration absorption peak of carbonyl group is at 1739 cm^−1^, and the absorption peak of C=C is at 1651 cm^−1^. It was established that to maximize the degree of crosslinking, the chemical bonding between HEMA and prepolymer was crucial to the synthesis of CWPU. Additionally, there is no absorption peak between 2000 and 2500 cm^−1^, which further suggests that the –NCO reaction is complete and results in the formation of the carbamate structure. The distinctive absorption peak of N–H in –CONH has a wave number at 1532 cm^−1^.

**Figure 2:**
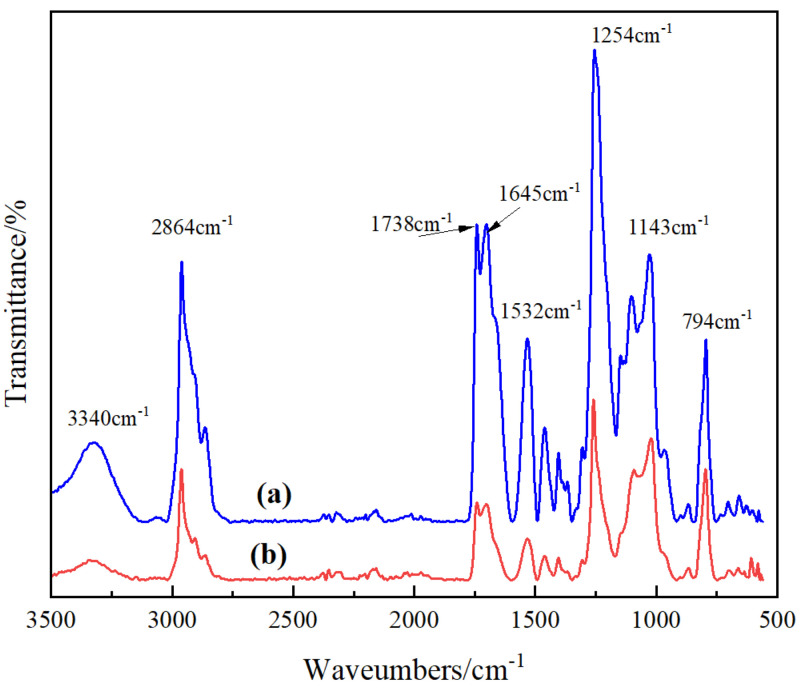
ATR spectrum of FPUA. (a) PA/PU = 50/50; (b) PA/PU = 30/70.

The curves (a) and (b) of Figure 2 depict the stretching vibration peaks of –NH– and –OH at 3340 cm^−1^, the methyl-methyl absorption peak at about 2910 cm^−1^ and 2864 cm^−1^, and the absence of any absorption peaks between 2000 and 2500 cm^−1^. It was demonstrated that throughout the synthesis process, –NCO underwent complete reactivity and changed into a carbamate structure. The carbonyl group has a stretching vibration peak at 1738 cm^−1^, a double bond has a characteristic absorption peak at 1645 cm^−1^, and N–H in –CONH has a characteristic absorption peak at 1532 cm^−1^, 1462 cm^−1^, and 1245 cm^−1^. The curve also reveals the deformation absorption peak of CF_2_ at 696 cm^−1^, the stretching vibration absorption peak of C–F at 1143 cm^−1^, the stretching vibration absorption peak of CF_3_ at 846 cm^−1^, and the absorption peak of CF_3_ at 794 cm^−1^, all of which signify the inclusion of fluorine in fluorinated polyurethane. Additionally, it can be seen from a comparison of curves (a) and (b) in Figure 2 that there is a variation in the strength of the same band peak when the PA/PU ratio is 50/50 and 30/70. The height of the 30/70 peak can be clearly seen to be higher than the 50/50 peak among them at 1738 cm^−1^ and 1532 cm^−1^, demonstrating that when PA/PU is 30/70, the absorption peak of the carbonyl group and carbamate bond is rather strong. Moreover, the absorption peak of 50/50 is stronger than that of 30/70 at 1143 cm^−1^ and 794 cm^−1^, further supporting the idea that when PA/PU is 50/50, the proportion of fluorine-containing acrylate increases, increasing the strength of the C–F and CF_3_ stretching vibration peak. This also further confirms the core-shell structure of the self-encapsulation behavior.

**Figure 3:**
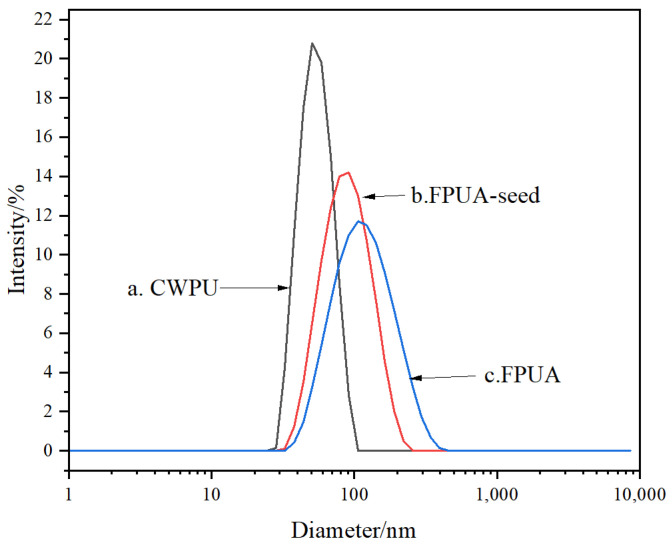
Particle size distribution. (a) CWPU; (b) 30 wt% FPUA; (c) 50 wt% FPUA.

Figure 3 shows that the particle size distributions of the CWPU and 30 wt% and 50 wt% FPUA emulsions are unimodal, indicating that the physicochemical distributions of the emulsions are fairly uniform. Among all the emulsions, PU has the smallest particle size and the narrowest particle size distribution, which indicates that the latex particles have good storage stability. Secondly, the modified 30 wt% FPUA emulsion had smaller particles and a narrower distribution compared to the 50 wt% FPUA emulsion. The amphiphilic CWPUs were self-assembled to form aggregates, which were surrounded by ionizable groups produced by the MDEA units. Relatively large aggregates were observed in FWPU-Seed solutions containing CWPU and one-third of the monomer. Considering the lack of hydrophilic portion of MMA and DFMA, it is assumed that these monomers migrate into the CWPU aggregates to reach a steady state and thus are responsible for the formation of larger aggregates. Water-soluble crosslinked DAAMs are dispersed on the surface of the polymer particles after polymerization to form chemical bonds and are susceptible to crosslinking during curing. In FWPU solutions containing CWPU and all monomers, the particle diameter further increases to nearly 100 nm.

**Figure 4:**
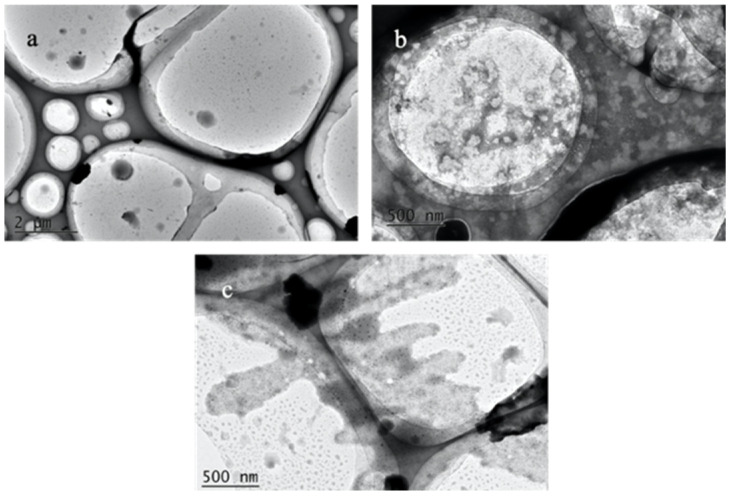
The TEM photograph of FPUA composite. (**a**) size of 2 μm; (**b**) size of 500 nm; (**c**) size of 500 nm.

Figure 4a,c depicts TEM images of FPUA emulsion particles demonstrating a core-to-shell ratio of 30/70 at particle sizes of 2 μm and 500 nm, respectively. For 500 nm sizes, Figure 4b portrays TEM images of FPUA emulsion particles with a core-to-shell ratio of 50/50. Initially, the TEM image exhibited a black-and-white pattern linked to light and dark upon negative staining of the emulsion with phosphotungstic acid. The polymeric particles were distinctly visible as spherical white spots on the dark background. An apparent core-shell architecture was discernible amongst them, with the hydrophilic PU chain segment functioning as the spherical shell of the particles and the hydrophobic fluoroacrylate monomer enveloped by PU forming the nucleus of particle. After comparison of TEM photographs of varied core-shell ratios, it was observed that in the 50/50 ratio of FPUA emulsion, numerous PA particles did not swell into PU for polymerization but copolymerized externally due to the incompatibility between the hydrophilic shell PU and hydrophobic PA. Consequently, the size of the core-shell’s internal and external halves was once diminished, simplifying the formation of a wrapped core-shell structure. However, a more uniform and distinguishable transtypic core-shell structure can be generated by FPUA emulsions exhibiting a core-to-shell ratio of 30/70.

**Figure 5:**
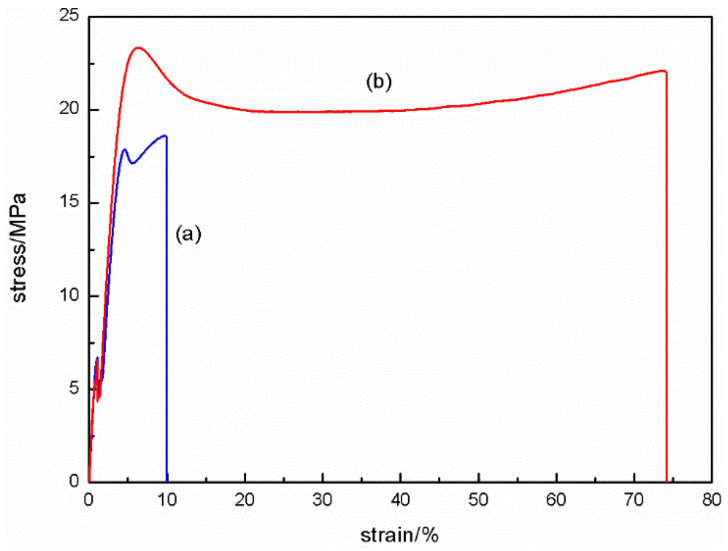
The tensile stress–strain curve of FPUA. (a) PA/PU = 50/50; (b) PA/PU = 30/70.

Figure 5 provides a graphical representation of how the core-shell ratio impacts the mechanical properties of latex film. For a more detailed insight into the data, refer to Table S2. The introduction of fluorine into the film increases its hardness, as is evident in the table. This is primarily due to the strong polar bond C–F in the fluorine-containing side chain of the hard segment of the polyurethane chain, which can generate NH^…^F hydrogen bonds. As the core-shell ratio increases, the tensile strength of the film initially increases before eventually decreasing. From 6.3 ± 0.05 to 23.35 ± 0.08 MPa, the tensile strength of FPUA rises. However, Young’s modulus and elongation at the break of the film gradually decline, indicating that as the core-shell ratio increases, the flexibility of the film decreases, making it more brittle. This observation highlights two key aspects. Firstly, fluorinated waterborne polyurethane has significantly higher hardness compared to unfluorinated polyurethane. However, it is less tough and flexible. Secondly, excessive fluorine content also impacts the mechanical properties of the film. When the core-shell ratio is increased to 50/50, the tensile strength of the film decreases from 23.35 ± 0.08 MPa to 18.61 ± 0.04 MPa. Alongside this, Young’s modulus reduces by 17.2%, while the elongation at break decreases by 86.5%. These findings indicate that as the proportion of fluorinated polyurethane adhesive (FPA) increases, the polarity of the composite emulsion gradually increases, leading to low compatibility between the hydrophilic group and the hydrophobic FPA segment. Consequently, the movement of the segment is inhibited, resulting in a significant reduction in flexibility and elongation at the break of FPUA and a softer more brittle film.

**Figure 6:**
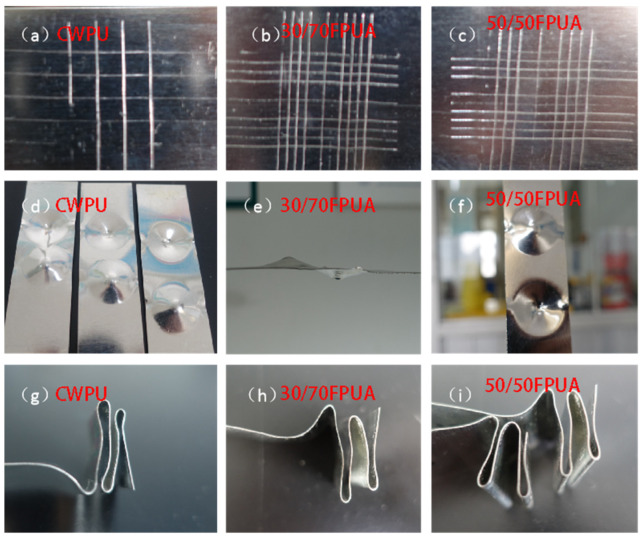
Adhesion (**a**–**c**), impact (**d**–**f**), and flexibility (**g**–**i**) tests of FPUA film.

The fluorine-containing acrylate modification of FPUA film yields notable enhancements in adhesion, impact resistance, hardness, and flexibility, as seen in Figure 6 and Table S3. This implies that the acrylate monomer addition raises the FPUA film’s crosslinking density, assisting in the formation of a dense network structure and improving the mechanical characteristics of the film.

**Figure 7:**
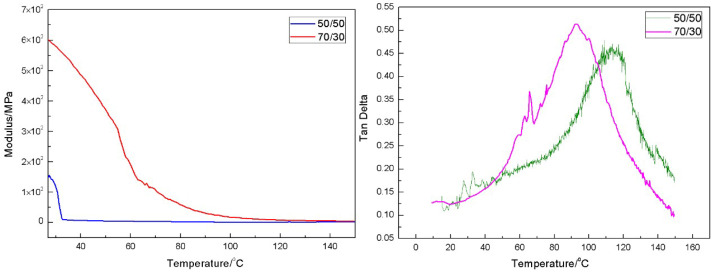
Storage modulus and tan δ for FPUA films.

Figure 7 demonstrates the relationship between mechanical loss (tan δ), energy storage modulus, and temperature for a cured FPUA film. Tan δ is a measure of the energy dissipation in a material under cyclic loading and its maximum value corresponds to the glass transition temperature (Tg) of the polymer. Tg represents the temperature at which the polymer transitions from a glassy state to a rubbery or viscous state. The modulus at Tg + 60 °C provides information about the cross-linking density of the film. Cross-linking density refers to the number of chemical bonds formed between polymer chains, which affects the stiffness and rigidity of the material. In this case, the cross-linking density and stiffness are directly related to both Tg and the modulus of the cross-linked polymer. Table S4 presents crucial data obtained from these calculations, specifically comparing the cross-linking densities of two different FPUA films: 50/50FPUA and 30/70FPUA. The results indicate that the cross-linking density of the 30/70FPUA film is significantly higher than that of the 50/50FPUA film, suggesting that the former film is more rigid. However, it is interesting to note that there are no significant differences in Tg and modulus between the 50/50FPUA and 30/70FPUA films in the glassy state. This implies that while the cross-linking density influences the rigidity of the films, it does not have a significant impact on their glass transition behavior. Therefore, the core-shell structure in this case manifests through variations in cross-linking density, which in turn affects the mechanical properties such as stiffness. The glass transition temperature and modulus serve as important parameters for understanding the behavior of these cross-linked polymer films.

**Figure 8:**
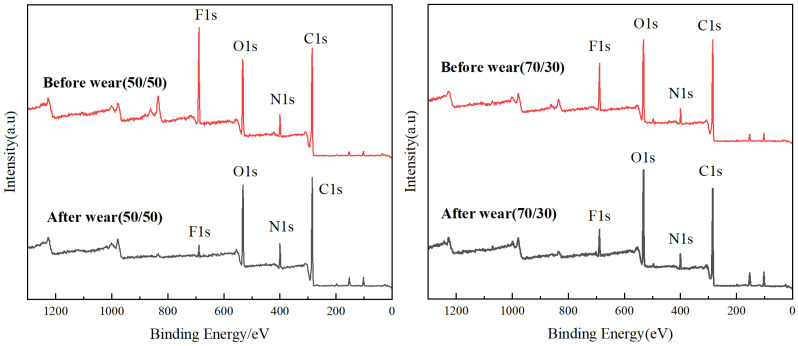
XPS wide spectra of the FPUA film.

Before and after wearing, the surface elements of the polished FPUA films with two core-shell ratios were investigated. As illustrated in Figure 8, the primary constituents on the surface of films are C, N, O, and F. Fluorine elements are more likely to migrate and enrich at the film/air surface following high-temperature heat treatment because their signal intensity is higher before grinding than after. Since the surface of the film is destroyed during grinding, the fluorine content decreases. Furthermore, comparing Figure 8, it can be shown that the enrichment of fluorine on the surface of the FPUA film is lower when the PA/PU ratio is 30/70 than when the PA/PU ratio is 50/50. Table S5 illustrates this quantitative growth more clearly.

**Figure 9:**
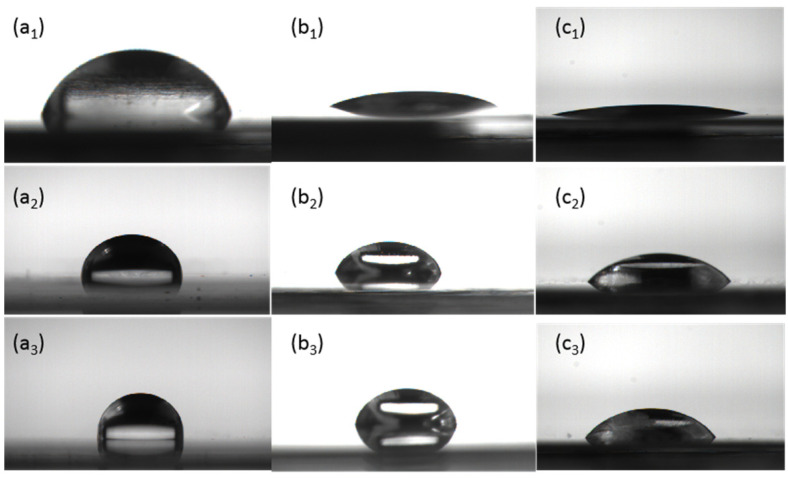
Images of droplets and the contact angles of water (**a_1_**–**a_3_**), diiodomethane (**b_1_**–**b_3_**), and hexadecane (**c_1_**–**c_3_**) on the coating.

The surface tension of the film is directly correlated with the magnitude of the contact angle. Typically, the lesser the contact angle and the lower the surface tension, the coarser the surface of film. Figure 9 and Table S6 demonstrate that, when specimens are moistened with identical solvent, the surface contact angle escalates in relation to fluoride content of samples. Owing to the strong electronegativity and diminished polarity of fluorine atoms, the structure of the backbone is cloaked in fluoro-bearing groups, which shield the interior hydrophilic groups of polymer molecules, thereby endowing the surface of the FPUA film with superior hydrophobic and oleophobic properties. The penetration capacity of various solvents into the surface of identical specimens also varies. Different solvents have different capacities for penetrating the surfaces of similar specimens. Hexadecane efficiently impregnates the sample’s surface more heavily than diiodomethane and water because of hexadecane’s lower polarity than water and its ability to deposit on the specimen surface at a moderate contact angle.

**Figure 10:**
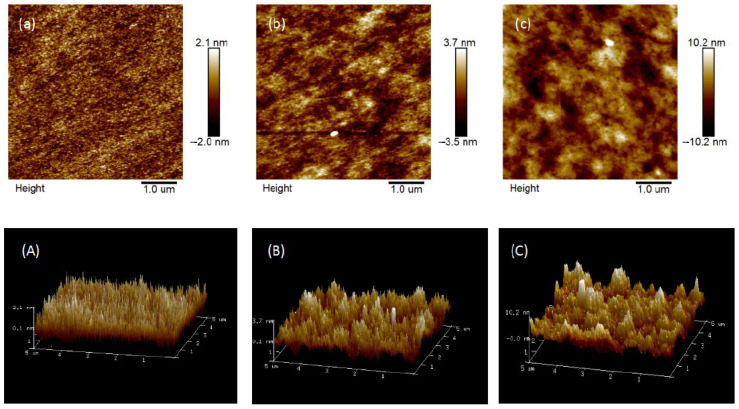
The AFM of CWPU/FPUA films. (**a**–**c**) CWPU/30 wt%/50 wt% FPUA; (**A**–**C**) CWPU/30 wt%/50 wt% FPUA.

Figure S1 shows the SEM of the coated film with a homogeneous surface without significant phase separation. Figure 10 shows the top view of the AFM plane of the coated film surface and its three-dimensional (3D) morphology image. From the figure, it can be observed that the phase region of the CWPU adhesive film in Figure 10A,a is small, relatively uniform, fluctuates within the range of 3 nm, and the surface flatness is high. On the other hand, the surface of fluorine-modified polyurethane is relatively high in roughness because of the incompatibility of the soft and hard segments, which will cause the phenomenon of micro-phase separation. Figure 10B,b,C,c shows the height map and 3D stereogram of FPUA gel film when the addition of FPA monomer is 30 wt% and 50 wt%, respectively. At 30 wt% FPA, the surface of the film is relatively flat and the roughness fluctuates around 7 nm, while at 50 wt% FPA, the surface of the film is the roughest and the fluctuation range is around 20 nm. This indicates that the fluorine content increases with the increase in the core-shell ratio and that the fluorine chain segments have the tendency to migrate to the air-adhesive film interface spontaneously during high-temperature curing, at this time, due to the low surface energy of fluorine; to reduce the surface area of the fluorine, it will form the bumps, which leads to the increase in surface roughness.

**Figure 11:**
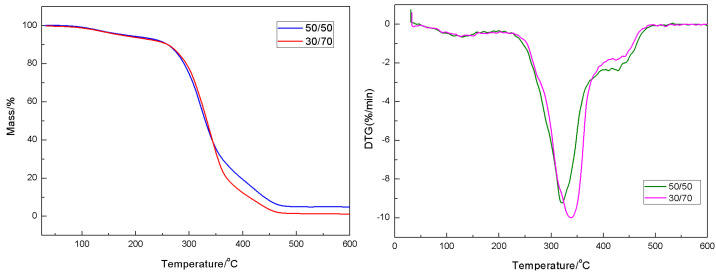
TGA curves and their corresponding DTG curves of FPUA films.

The TGA curves of FPUA-coated films in nitrogen and their thermogravimetric analyses (DTG) are shown in Figure 11, respectively. The residual carbon content (Y_c_) and the temperature of maximum loss rate (T_max_) of the samples after 600 °C are shown in Table S7. The thermal decomposition temperatures of the polymers do not differ significantly at the initial stage and at 20% weight loss, the decomposition temperatures of the two are comparable, even the decomposition temperature of 50/50FPUA is slightly lower than that of 30/70FPUA, which may be attributed to the poor thermal stability of the MMA chain segments. The subsequent decomposition process can be divided into two main stages, corresponding to the thermal decomposition of the soft and hard phases of the polymer. The decomposition temperature of the hard phase of 50/50FPUA is 254 °C (the C–N bonding energy of the hard phase is low) and the decomposition end temperature is 393 °C, with the maximum decomposition rate occurring at 320.1 °C, while the initial decomposition temperature of the soft phase is 410 °C and the decomposition end temperature is 475 °C. The decomposition stages of the soft and hard phases of 30/70 are not much different from those of 50/50FPUA but the maximum decomposition rate is greater than that of 30/70FPUA and the maximum decomposition rate is higher than that of 50/70FPUA, which is more obvious. The decomposition stage of the 30/70 soft and hard phases is similar to that of the 30/70 phase but the maximum decomposition rate is greater than that of the 50/50 FPUA, which shows that the heat resistance of FPUA increases with the increase in the proportion of FPA. This is because the addition of fluorinated monomers further improves the heat resistance of the coating film due to the larger bond energy and higher stability of the C–F bond. The higher the DFMA content in the shell layer in the core-shell structure, the higher the C–F bond binding energy, the more stable the structure, and the better the heat resistance.

**Figure 12:**
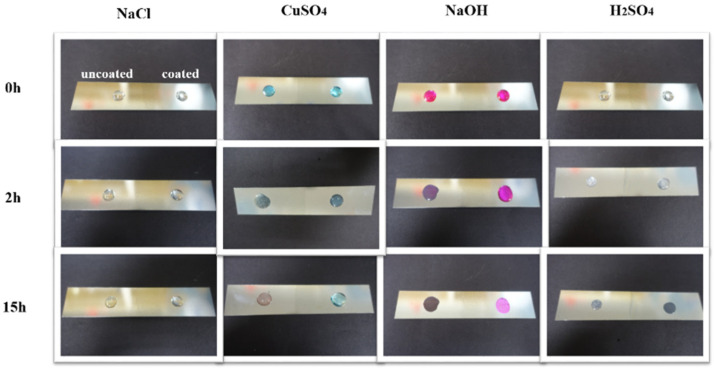
Corrosion resistance of FPUA films.

The bulk of the uncoated tinplates have corroded to discoloration because of a chemical reaction between the solution on their surface and the iron sheet substrate, as seen in Figure 12. The tinplate surface coated with FPUA emulsion, however, still maintains high surface integrity and very little corrosion after 15 h of acid–alkali salt corrosion. This demonstrates that the modified FPUA film has good corrosion resistance and can withstand the interference of a harsh external environment.

**Figure 13:**
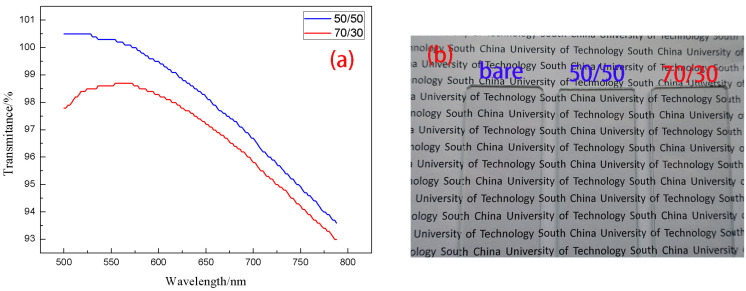
Transmittance test of FPUA film. (**a**) Transmittance Curve; (**b**) Corresponding coatings are tested for transparency.

The transmittance curves for different light wavelengths are shown in Figure 13 after the glass plate has been coated. As the wavelength of the incoming light increases, the transmittance of the film shows a trend of slight rise at first, followed by a reduction, as seen in Figure 13a. Under the two ratios, the transmittance of film in the visible spectrum (380–780 nm) is greater than 93%. The quantity of light passing through the slide film displaying the clear and transparent text before and after coating was found to be negligible to nonexistent in natural light, as shown in Figure 13b. This reveals that the system is extremely stable and that the particle size of the emulsion is consistent. It also shows that the produced FPUA emulsion has a high degree of transparency and a smooth granular-free film. It is crucial for electrical and film goods that have to adhere to stringent transparency standards.

## 4. Conclusions

In summary, a core-shell fluorinated polyurethane-acrylate composite emulsion was prepared by using synthetic cationic waterborne polyurethane as the seed emulsion and pre-emulsions were made by solubilizing with acrylic monomers, such as DFMA, MMA, and DMMA, to prepare a core-shell fluorinated polyurethane-acrylate composite emulsion; then, ADH was introduced as the post-crosslinking agent and keto hydrazine cross-linking was used to make the polymer form a chemically crosslinked film between the core-shell and the core-shell. The experiments mainly investigated the effects of fluorinated acrylate modification before and after the modification as well as the effects of two different core-shell ratios, 30/70 and 50/50, on the properties of FPUA emulsions and adhesive films. The results showed that the emulsion storage stability was better when the addition of fluorinated acrylic acid (PA) was in the range of 30–50 wt%. It was observed by TEM that the polymer particles showed a clearly visible core-shell structure, with the hydrophilic PU chain segments as the shell and the hydrophobic PA chain segments as the core. After high temperature heat treatment, the fluorine content on the surface of FPUA film increases with the increase in the core-shell ratio, up to 14.75%, which is also verified by the AFM test of the film. The contact angle test on the surface of the film shows that when w(PA) is 50%, the surface contact angle reaches 98.5°, which is hydrophobic to a certain extent. The tensile strength of the films was found to increase and then decrease with the increase in the core-shell ratio and the maximum was 23.35 ± 0.08 MPa at a core-shell ratio of 30/70; however, with the increase in the core-shell ratio to 50/50, the number of fluorine-containing chain segments increased and the density of the polar groups became larger and larger, which impeded the free movement of the chain segments and thus made the FPUA films hard and brittle. With the increase in the core-shell ratio, the heat resistance of FPUA films gradually increased. The fluorinated acrylate-modified FPUA film has excellent adhesion, impact resistance, hardness, flexibility, and chemical resistance. The light transmittance of the prepared films is above 93%, which has high application value. In the future, we will conduct in-depth research on the process of fluorinated acrylate-modified cationic waterborne polyurethanes, including the effective promotion of the compatibility of the two phases of PU and PA, the enhancement of the conversion rate of fluorinated chain segments, and the reduction in process costs.

## Data Availability

The data are contained within the article.

## Supplementary Materials

The following supporting information can be downloaded at: https://www.mdpi.com/article/10.3390/polym16010086/s1, Figure S1: SEM images of the films. (a) Monocrystalline silicon coated sheet; (b) CWPU; (c) 30/70FPUA; (d) 50/50 FPUA; Table S1: Typical dosage for cationic polyurethane aqueous dispersion (CWPU); Table S2: Tensile strength, Elongation at break and Young’s modulus of FPUA; Table S3: The adhesion, impact resistance and flexibility of FPUA; Table S4: The T_g_, modulus and crosslink density of FPUA; Table S5: Atomic percentage of FPUA film surfaces before and after wearing test; Table S6: The contact angle of water, diiodomethane and hexadecane droplets on the films; Table S7: Thermal stability of FPUA films.
