# Supplementary material for: Synthesis and Properties of Cationic Core-Shell Fluorinated Polyurethane Acrylate
Source: Polymers (Basel). 2023 Dec 27;16(1):86. doi: 10.3390/polym16010086 (PMC10780964; doi:10.3390/polym16010086)
Supplement: Supplementary file 1 [file polymers-16-00086-s001.zip › polymers-2784034-supplementary.pdf]

Synthesis and properties of cationic core-shell fluorinated polyurethane acrylate

Junhua Chen [1,2], Xiaoting Lu [1], Jinlian Chen [1], Shiting Li [1], He Zhang [1,2], Yinping Wu [1,2], Dong Yu Zhu [3], Xiangying Hao [1,2*]

[1] School of Environmental and Chemical Engineering, Zhaoqing University, Zhaoqing 526061, China;

[2] Guangdong Provincial Key Laboratory of Environmental Health and Land Resource, College of Environmental and Chemical Engineering, Zhaoqing University, Zhaoqing 526061, PR China;

[3] School of Chemical Engineering and Light Industry, Guangdong University of Technology, Guangzhou 510006, China

Table S1. Typical dosage for cationic polyurethane aqueous dispersion (CWPU)

IPDI/ mol	PCDL/mol	MDEA /mmol	TMP/ mmol	BDO/ mmol	HEMA/ mol
0.07	1.75×10^{-2}	2.9	4.4	4.4	1.2×10^{-2}

Table S2. Tensile strength, Elongation at break and Young's modulus of FPUA

**Samples**	**Tensile strength/MPa**	**Elongation at break/%**	**Young's modulus/MPa**
**CWPU**	6.3±0.05	108.21	724.62±0.05
**30/70FPUA**	23.35±0.08	74.17	680.88±0.08
**50/50FPUA**	18.61±0.04	9.98	563.96±0.04

Table S3. The adhesion, impact resistance and flexibility of FPUA

**Samples**	**Pencil hardness**	**Adhesion/level**	**Flexibility/mm**	**Impact resistance**
**CWPU**	H	2	1	Undamaged
**30/70 FPUA**	2H	1	1	Undamaged
**50/50FPUA**	2H	1	1	Undamaged

Table S4. The T_g, modulus and crosslink density of FPUA

**Samples**	**T_g/°C**	**Modulus at 25°C/MPa**	**Modulus at T_g +60°C/MPa**	**Crosslinking density/mol ▪m^{-3}**
**50/50FPUA**	113.6	150	2.6	233.4
**30/70FPUA**	92.9	600	5.4	508.3

Table S5. Atomic percentage of FPUA film surfaces before and after wearing test.

**Samples***	**F**	**O**	**N**	**C**
a_1	14.75%	18.32%	7.21%	59.72%
a_2	2.56%	20.96%	8.87%	67.6%

b_1	9.31%	21.58%	6.19%	62.92%
b_2	5.47%	25.43%	6.45%	62.65%

* (a_1, a_2, b_1, b_2 represent the elemental content before and after PA/PU=50/50; PA/PU=30/70 film wearing, respectively.)

Table S6. The contact angle of water, diiodomethane and hexadecane droplets on the films

Test droplets	Water	Diiodomethane	Hexadecane
**CWPU**	68°	22°	15°
**30/70FPUA**	89°	66°	51°
**50/50FPUA**	99°	74°	55°

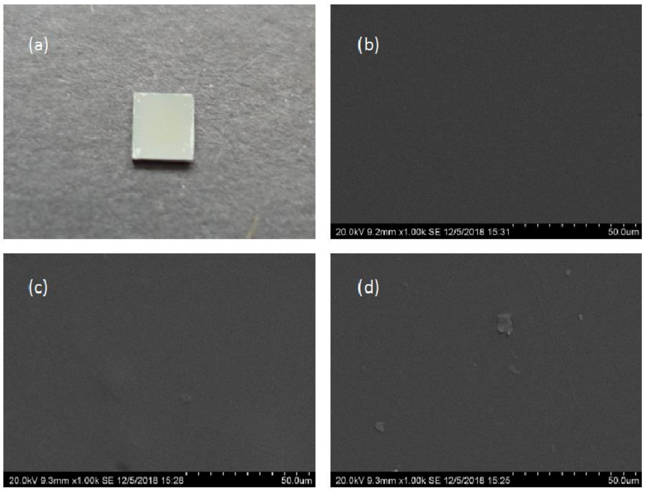

Figure S1. SEM images of the films. (a) Monocrystalline silicon coated sheet;(b) CWPU; (c)30/70FPUA; (d)50/50 FPUA

The SEM micrograph depiction of the CWPU, 30/70FPUA, and 50/50FPUA films, respectively, on the film surface are illustrated in Figures S1(b), (c), and (d). While both 30/70FPUA and 50/50FPUA film surfaces exhibit varying levels of minor protuberances, which are instigated by the incompatibility of fluorinated groups and the polyurethane carbon chain due to the soft and hard bimodal micro-phase segregation phenomena, the WPU film surface possesses the most smooth and planar character among the group. Fluorinated groups will migrate to the surface of film during the formation of dry films, which would accentuate the micro-phase separation phenomena. Nevertheless, the macro structure remains unaltered as a whole, notwithstanding the presence of some level of separation at the micro level.

Table S8. Thermal stability of FPUA films

Formulation	T_{max1} (°C)	T_{max2} (°C)	Y_c at 600 °C (%)
50/50FPUA	337	418	4.95
30/70FPUA	339	428	1.26
